# A Porous Polymer-Based Solid Acid Catalyst with Excellent Amphiphilicity: An Active and Environmentally Friendly Catalyst for the Hydration of Alkynes

**DOI:** 10.3390/polym11122091

**Published:** 2019-12-13

**Authors:** Yizhu Lei, Maomin Zhang, Qian Li, Yu Xia, Guojun Leng

**Affiliations:** School of Chemistry and Materials Engineering, Liupanshui Normal University, Liupanshui 553004, China; zhangmaomin0905@163.com (M.Z.); qliscir@163.com (Q.L.); L1020XiaYu@163.com (Y.X.); guojunlps@126.com (G.L.)

**Keywords:** porous organic polymer, solid acid, amphiphilicity, water

## Abstract

Developing efficient solid acid catalysts for aqueous organic reactions is of great importance for the development of sustainable chemistry. In this work, a porous polymeric acid catalyst was synthesized via a solvothermal copolymerization and a successive ion-exchange method. Physicochemical characterizations suggested that the prepared polymers possessed large Brunauer-Emmett-Teller (BET) surface areas, a hierarchically porous structure, excellent surface amphiphilicity, and nice swelling properties. Notably, an activity test in phenylacetylene hydration indicated that the prepared solid acid exhibited high catalytic activity in water, which outperformed commercial amberlyst-15, sulfuric acid, and benzenesulfonic acid. Moreover, the prepared solid acid can be easily recovered and reused at least four times. Additionally, a variety of aromatic and aliphatic alkynes could be effectively transformed into corresponding ketones under optimal reaction conditions.

## 1. Introduction

The increasing environmental concerns about harmful solvent waste has led to a considerable interest in using water as a solvent for synthetic organic chemistry [1,2]. Brønsted acids have been widely used in many water-mediated organic syntheses, such as hydrolysis and hydration reactions [3,4,5]. However, performing an organic reaction with liquid Brønsted acid suffers from inherent drawbacks, such as strong corrosivity and high-volume wastes [6,7]. Towards this end, heterogeneous switching of homogeneous Brønsted acids has been developed and attracted a lot of interest [8,9,10].

The hydration of alkynes represents one of the most straightforward and efficient ways for the synthesis of carbonyl compounds [11]. The traditional reaction system over a catalytic amount of mercury salts has been known for more than a century. However, mercury salts are highly toxic. Therefore, many low-toxic metal catalysts, such as Au [12,13,14], Ag [15,16], Pt [17,18], and Ru [19] salts, as well as Brønsted acid (e.g., trifluoromethanesulfonic acid (TfOH), Triflimide (HNTf_2_) catalysts have been developed for replacing the toxic mercury salts [20,21]. Among these catalysts, liquid Brønsted acids are inexpensive, and to some extent, environmentally friendly [20]. Therefore, they are more appealing for practical application. However, the inherent drawbacks, such as strong corrosivity, high-volume wastes, and difficulty in separating and recycling, limit their practical applications. Hence, it is desirable to develop highly efficient and water-compatible solid acid catalysts for alkyne hydration.

Porous organic polymers (POPs), which feature designable chemical functionalities and large surface areas, have recently received great research interest in the field of catalysis, due to their potential to combine the advantages of heterogeneous and homogeneous catalysis [22,23,24,25]. To date, a considerable number of porous polymeric acids have been successfully developed for various acid-catalyzed reactions [26,27,28]. Generally, for solid catalysts in water, the catalysts should be designed to be amphiphilic, thus allowing the nice contact between organic substrates, water, and the solid catalyst [29,30,31]. However, most porous polymeric acids are mainly composed of aromatic frameworks, endowing the catalyst with hydrophobic surface wettability [26,27,32], and thus, restricting their catalytic applications in water. Porous ionic polymers (PiPs) [33,34], which represent a new kind of porous organic polymer (POP) [33,34,35], could be easily prepared from the free radical polymerization of vinyl-functionalized organic salts. When used as the catalytic supports, PiPs can exhibit not only the features of POPs, but also some additional advantages, such as allowing easy functionalization via ion-exchange reactions and easy adjustment of surface wettability of the polymer [35,36,37,38].

**Scheme 1:**
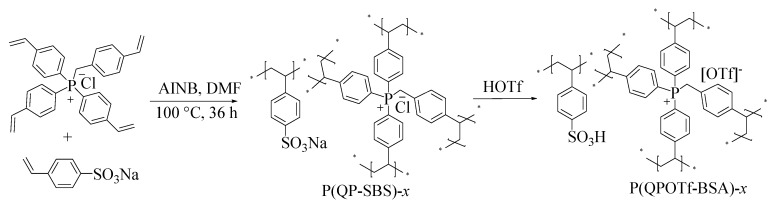
Preparation of the solid acid catalysts.

To develop an efficient and solid acid catalyst for the hydration of alkynes in water, herein, we initiate the synthesis of amphiphilic porous polymeric acids containing both the sulfonic and phosphonium salt groups (P(QPOTf-BSA)) and study their catalytic performances in water- mediated alkyne hydration. The amphiphilic and hyper-cross-linked P(QPOTf-BSA) was facilely synthesized through the free-radical copolymerization of 4-vinylbenzyl-tris-(4-vinylphenyl)- phosphonium chloride (QP) and sodium p-styrene sulfonate, followed by ion-exchange with HSO_3_CF_3_, as shown in Scheme 1. An activity test in phenylacetylene hydration suggested that the obtained P(QPOTf-BSA) exhibited excellent activity, outperforming the activities of heterogeneous amberlyst-15, as well as homogeneous sulfuric acid and benzenesulfonic acid. Furthermore, P(QPOTf-BSA) could be easily recovered and reused at least four times.

## 2. Materials and Methods

### 2.1. Materials

4-Vinylbenzyl chloride (90%), sodium p-styrene sulfonate (SBS), 2,2′-azobis(2-methylpropionitrile) (AIBN), alkynes, *N,N*-dimethylformamide, and benzenesulfonic acid were supplied by Energy Chemical Company (Shanghai, China). Amberlyst-15 and trifluoromethanesulfonic acid (HOTf) were purchased from Sigma-Aldrich Company (Shanghai, China). Sulfuric acid (98%) was obtained from Tianjin Guangfu Chemical Reagent (Tianjin, China). Tris(4-vinylphenyl)phosphine (TVP) was prepared as our previous report [39]. 4-Vinylbenzyl-tris-(4-vinylphenyl)-phosphonium chloride (QP) was prepared by a simple reaction of TVP and 4-vinylbenzyl chloride [40]. QP, ^1^H NMR (400 MHz, CDCl_3_): *δ* = 7.66–7.50 (m, 12H), 7.08–7.02 (m, 4H), 6.69–6.62 (m, 3H), 6.61–6.57 (m, 1H), 5.85 (d, *J* = 17.6 Hz, 3H), 5.59 (d, *J* = 17.6 Hz, 1H), 5.43–5.38 (m, 5H), 5.15 (d, *J* = 10.9 Hz, 1H) ppm.

### 2.2. Catalyst Preparation

SO_3_Na-functionalized porous ionic polymer, P(QP-SBS)-x, where x refers to the mole fraction of SBS monomer to the total monomers, was solvothermally synthesized via the copolymerization of SBS and QP. Taking the preparation of P(QP-SBS)-0.5 as an example, QP (1.41 g, 2.86 mmol), SBS (0.59 g, 2.86 mmol), and AIBN (50 mg) were dissolved in 20 mL DMF in a 100 mL autoclave. After replacing air in the autoclave with nitrogen, the mixture was magnetically stirred at room temperature for 2 h. Then, the autoclave was heated in an oven at 100 °C for 24 h. After cooling to room temperature, the resulting monolith solid was extracted with ethanol and dried in vacuum. Thus, P(QP-SBS)-0.5 (2.03 g) was obtained as a white solid. Changing the mole fraction of SBS and keeping the total mass of the monomers at 2.0 g, the other two samples, P(QP-SBS)-0.25 and P(QP-SBS)-0.75, were also prepared according to the above procedures. Furthermore, the prepared P(QP-SBS)-x samples were treated by ionic exchange using a solution containing 50 mL of toluene and 5 mL of HSO_3_CF_3_ for 12 h at room temperature. After repeating the ionic exchange treatment three times, the solid was filtered, washed with CH_2_Cl_2_ and water, and then dried under vacuum (60 °C). After grinding in agate mortar, a light grey solid was obtained and donated as P(QPOTf-BSA)-x. The acid amount of the sample was measured by a reverse acid–base titration method using 0.1 M HCl solution. Specifically, 2 mL of NaOH (1 M) was added to 0.2 g of catalyst. After stirring at room temperature for 1 h, the solution was filtered and washed with deionized water until the filtrate was neutral. Then, the filtered solution was titrated with HCl using phenolphthalein as an indicator.

### 2.3. Catalytic Activity

The synthesis of ketones via alkyne hydration was conducted in a 10 mL pressure-resistant reaction tube. Typically, alkyne (2.0 mmol) was added to 4 mL of deionized water in the reaction tube. Then, the P(QPOTf-BSA)-0.5 (H+, 15 mol%; 0.256 g) powder was added into the mixture. The resulting mixture was heated in a preheated oil bath at 120 °C for 7 h. After the reaction, the mixture was separated by centrifugation and the obtained liquid was quantitatively analyzed on gas chromatography (GC) using *N,N*-dimethylacetamide as an internal standard. For the recycling experiments, the recovered catalyst was washed with ethanol and water, and then used directly for the next catalytic run.

### 2.4. Adsorption Capacity

Adsorption capacities of P(QPOTf-BSA)-x samples for phenyl acetylene were measured in the H_2_O-phenyl acetylene mixture. Generally, P(QPOTf-BSA)-x (0.15 g) was added into the mixture containing water (5.0 g) and phenyl acetylene (2.0 g). After stirring at room temperature for 30 min, the mixture was separated by centrifugation. The solid was extracted with ethanol, and the resulting liquid was quantitatively analyzed on gas chromatography (GC).

### 2.5. Characterization

The N_2_ adsorption and pore size distribution were recorded on a Micrometrics ASAP 2020 ((Micromeritics Instrument Co., Norcross, GA, USA) at 77 K. Scanning electron microscopy (SEM and SEM-mapping images were viewed on a Hitachi-S4800 instrument (Hitachi Ltd., Tokyo, Japan). Transmission electron microscopy (TEM) images were collected on a Philips Tecnai G2 F30 instrument (Philips-FEI Co., Amsterdam, Netherlands). FT-IR spectroscopy was recorded on a Bruker Equinox 55 FT-IR spectrophotometer (Bruker Corp., Rheinstetten, Germany). ^13^C and ^31^P solid-state NMR experiments were performed on a Bruker AVANCE III 600 Bruker spectrometer (Bruker Corp., Rheinstetten, Germany). X-ray photoelectron spectroscopy (XPS) measurements were carried out on a VG MultiLab 2000 X-ray photoelectron spectrometer (Thermo Electron Corporation, Waltham, MA, USA). The thermogravimetric analysis (TGA) was measured on a NETZSCH STA 449 F5 instrument (Netzsch, Serb, Germany) under a dynamic argon atmosphere, and the sample was analyzed from room temperature to 600 °C with a heating rate of 10 K min^−1^. Contact angle measurement was performed on a DataPhysics OCA 20 contact angle system (Data-Physics, Filderstadt, Germany). Quantitative analysis of the reaction liquid was performed on a gas chromatograph (Scientific™ TRACE™ 1310, Thermo Electron Corporation, Waltham, MA, USA) equipped with a FID detector and a capillary column (TRACE TR-WAX, Thermo Electron Corporation, Waltham, MA, USA).

## 3. Results

### 3.1. Characterization

P(QPOTf-BSA)-x, where x refers to the mole fraction of SBS to the total monomers, were successfully prepared in quantitative yields via the solvothermal copolymerization of QP and SBS, followed by ion-exchange with HSO_3_CF_3_. The structure and properties of the prepared samples were characterized by N_2_ adsorption–desorption, SEM, TEM, FT-IR, XPS, solid-state ^13^C NMR spectra, SEM-mapping, and contact angle measurement.

**Figure 1:**
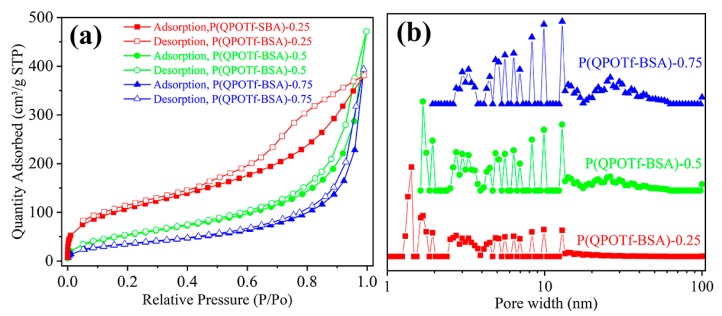
N_2_ absorption–desorption isotherms (**a**) and pore size distribution curves (**b**) of the P(QPOTf-BSA)-*x* samples.

The surface areas, pore volumes and pore radius of P(QPOTf-BSA)-x samples are shown in Table 1. BET surface areas of P(QPOTf-BSA)-0.25, P(QPOTf-BSA)-0.5, and P(QPOTf-BSA)-0.75 were 392, 204, and 130 m^2^/g, respectively. With the increment of the mole fraction of the SBS monomer, surface areas of the samples decreased gradually. This could be reasonably assigned to the decreased cross-linking degrees of the polymer frameworks. The N_2_ adsorption–desorption isotherms of the samples are depicted in Figure 1a. The three samples featured the combined sorption behavior of type I and type IV by International Union of Pure and Applied Chemistry (IUPAC) classifications. The adsorption behavior of the three samples below P/P_0_ < 0.01 suggested that P(QPOTf-BSA)-0.25 had more micropores than P(QPOTf-BSA)-0.50, and P(QPOTf-BSA)-0.75 had the least. While the slopes in the regions of P/P_0_ = 0.8–1.0 revealed that P(QPOTf-BSA)-0.25 had less macropores than P(QPOTf-BSA)-0.50, and P(QPOTf-BSA)-0.75 had the most. All of the three samples showed the hysteresis loops at P/P_0_ in the range 0.7–1.0, reflecting the presence of mesopores. These results were consistent with the pore size distribution curves of the samples, as shown in Figure 1b. P(QPOTf-BSA)-0.25 mainly consisted of micropores and mesopores, while P(QPOTf-BSA)-0.5 and P(QPOTf-BSA)-0.75 had the broader pore size distribution, comprising of micropores, mesopores, and macropores.

**Figure 2:**
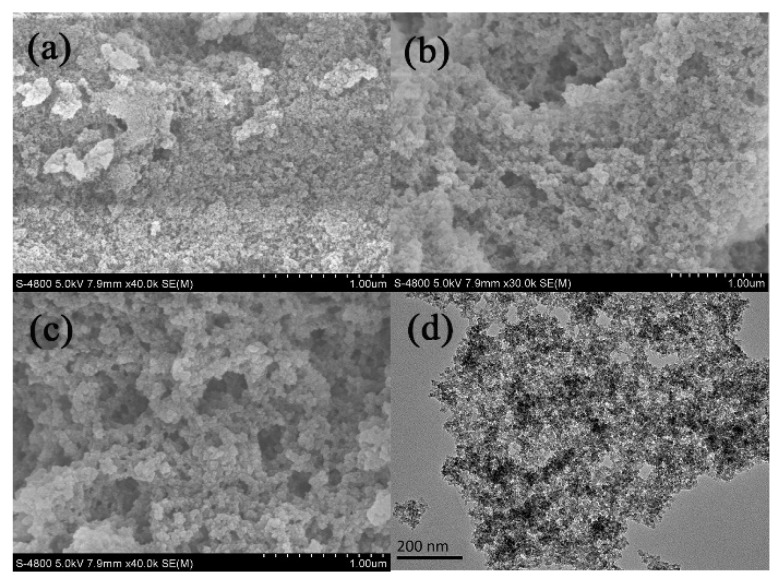
SEM images of the prepared samples: (**a**) P(QPOTf-BSA)-0.25, (**b**) P(QPOTf-BSA)-0.5, (**c**) P(QPOTf-BSA)-0.75, (**d**) TEM image of P(QPOTf-BSA)-0.5.

SEM analysis was used to view the morphology of P(QPOTf-BSA)-*x* samples. The results, as shown in Figure 2a–c, suggested that the samples showed an irregular porous structure that was comprised of small particles. TEM images, as shown in Figure 2d, of the representative P(QPOTf-BSA)-0.5 sample confirmed the existence of mesopores between the agglomerated small particles. Thus, SEM and TEM images further proved the existence of hierarchical porosities. For the solid catalyst, the hierarchical pore structure with abundant micropores and mesopores would be beneficial for heterogeneous catalysis by accelerating the mass transport of reactants and products [41].

**Figure 3:**
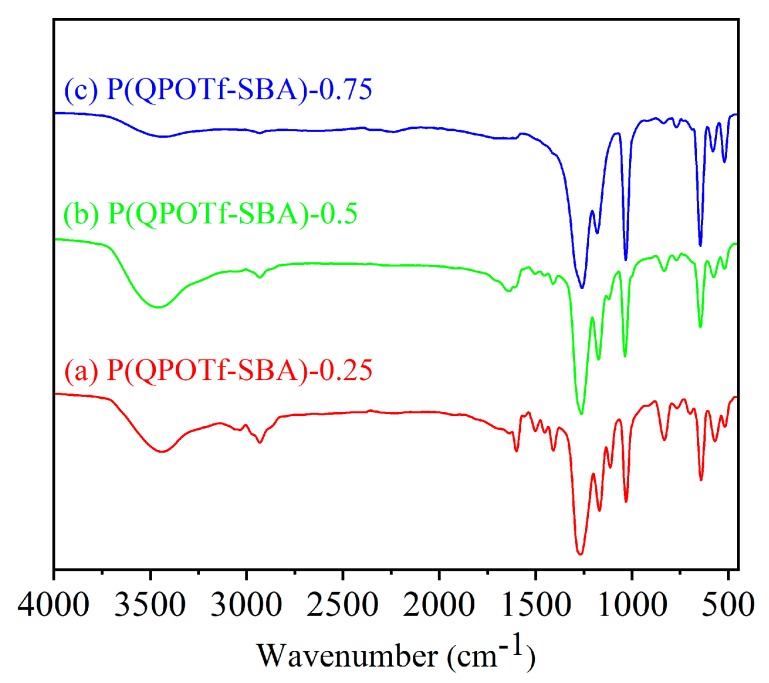
FT-IR spectra of P(QPOTf-BSA) samples.

**Figure 4:**
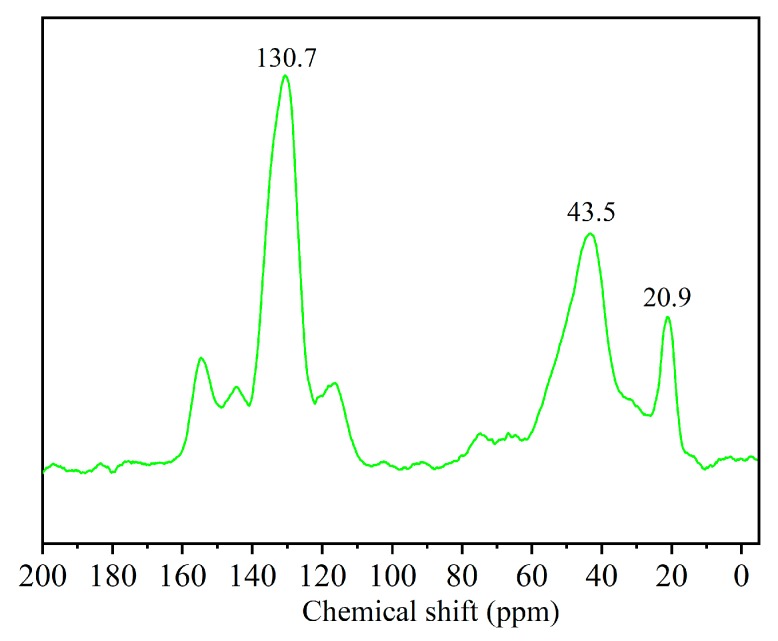
Solid-state ^13^C NMR spectrum of P(QPOTf-BSA)-0.5.

FT-IR spectra of P(QPOTf-BSA)-*x* samples are shown in Figure 3. The three samples showed similar absorption bands, indicating their analogous composition. The vibration at around 1260 and 1034 cm^−1^ could be associated with the S=O and C–S vibrations [28]. The peaks at 1175 cm^−1^ could be attributed to the C–F and S–O vibrations [28,42]. Figure 4 showed the ^13^C solid-state NMR spectrum of a representative sample of P(QPOTf-BSA)-0.5. The signal at around 130.7 ppm associated with the aromatic carbons could be observed [40,43]. The peak at 43.5 ppm associated with the polymerized vinyl groups could also be observed [40,43]. The solid-state ^31^P NMR spectrum of P(QPOTf-BSA)-0.5 displayed a single peak at around 21 ppm, as shown in Figure S1 from Supplementary Materials. This peak could be assignable to phosphonium salt in the framework [40]. Thus, these results verified the successful preparation of the polymer samples containing sulfonate groups.

**Figure 5:**
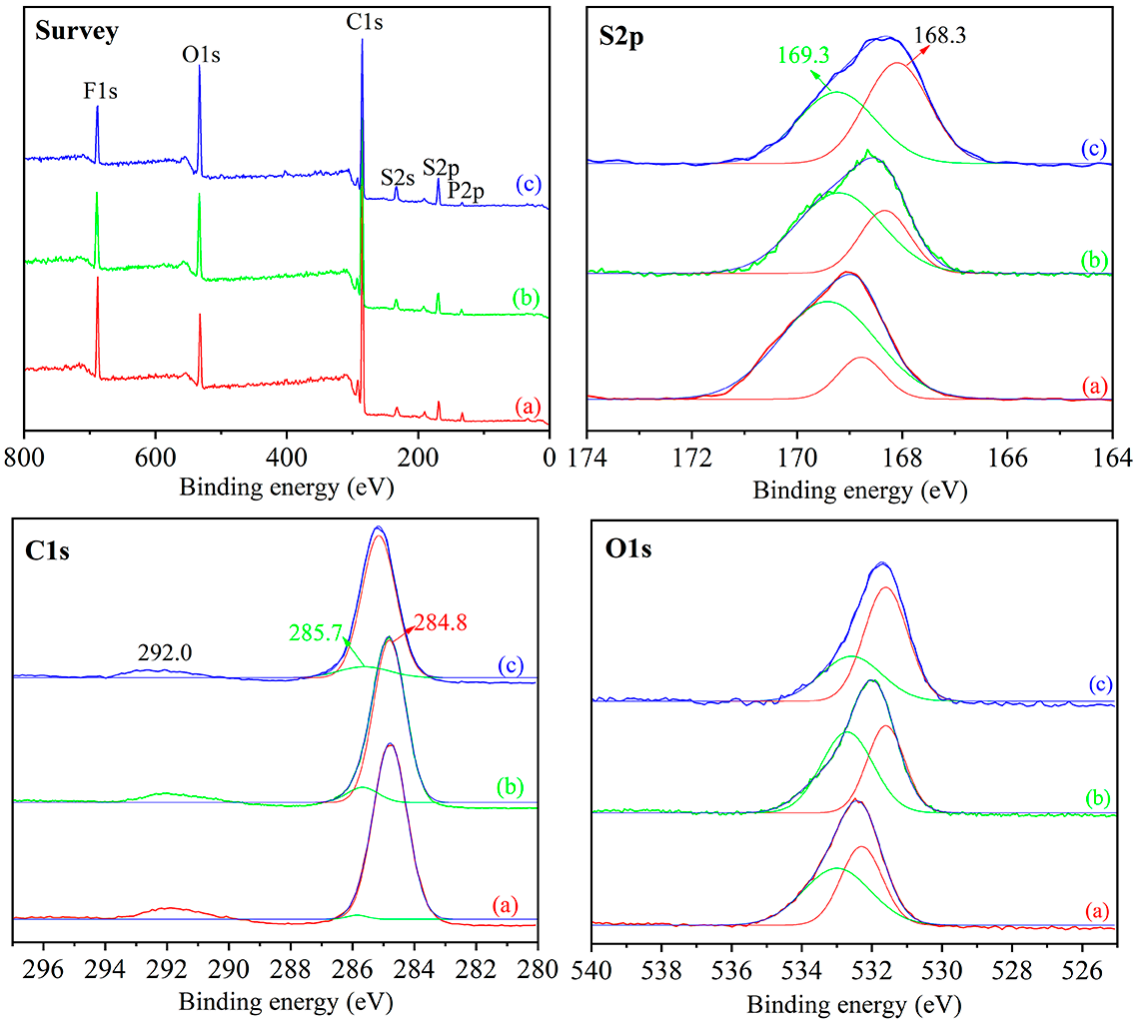
X-ray photoelectron spectroscopy (XPS) spectra of the prepared samples: (**a**) P(QPOTf-BSA)-0.25, (**b**) P(QPOTf-BSA)-0.5, (**c**) P(QPOTf-BSA)-0.75.

**Figure 6:**
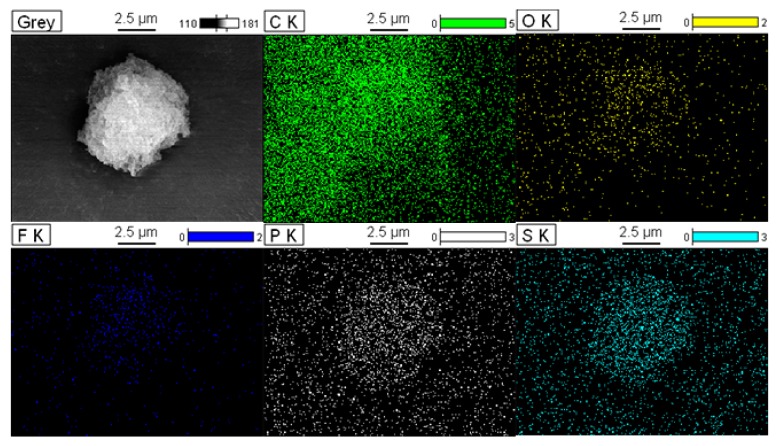
SEM-mapping analysis of P(QPOTf-BSA)-0.5.

Figure 5 shows X-ray photoelectron spectroscopy (XPS) of the prepared samples. The XPS full spectra in Figure 5a confirmed the presence of C, O, P, S, and F elements, and the absence of Na and Cl elements in the three samples, suggesting that the ion exchange between P(QP-SBS)-*x* and CF_3_SO_3_H was finished. The S2p peaks at around 168.3 and 169.3 eV associated with the sulfur atoms of –SO_3_H and CF_3_SO_3_¯ groups could be observed [26,44]. The signals of C1s peaks at around 284.8, 285.7, and 292.0 eV were attributed to C–C, C–S, and C–F bonds, respectively [26]. The O1s peaks at around 531.6 and 532.7 eV were assigned to the oxygen atoms of –SO_3_H and OTf¯ groups [45]. These results further confirmed the successful introduction of –SO_3_H and OTf¯ groups in the P(QPOTf-BSA) samples. The element distribution of a representative sample of P(QPOTf-BSA)-0.5 was characterized by SEM-mapping analysis. As depicted in Figure 6, the O, F, P, and S elements were clearly observed and well-distributed in the representative sample. Thermogravimetric analyses (TGA) of the representative P(QPOTf-BSA)-0.5 sample, as shown in Figure S2 from Supplementary Materials, showed that the main weight loss occurred above 200 °C, suggesting that the prepared samples could be stable up to 200 °C. A little weight loss around 4 wt % could be also observed below 100 °C; this weight loss could be mainly ascribed to the removal of trapped guest molecules, which is common for porous materials.

**Figure 7:**
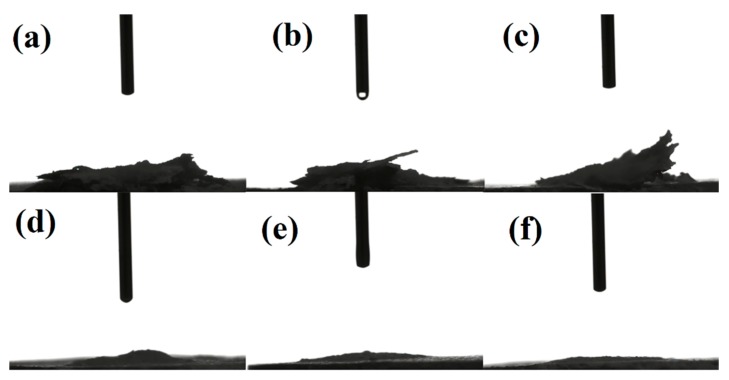
Images of contact angle measurements: (**a**) P(QPOTf-BSA)-0.25 for water, (**b**) P(QPOTf-BSA)-0.5 for water, (**c**) P(QPOTf-BSA)-0.75 for water; (**d**) P(QPOTf-BSA)-0.25 for phenyl acetylene, (**e**) P(QPOTf-BSA)-0.5 for phenyl acetylene, (**f**) P(QPOTf-BSA)-0.75 for phenyl acetylene.

For catalytic reactions in water with hydrophobic organic substrates, surface wettability of the solid catalyst is vitally important for achieving high catalytic performance [29,30,31]. To test the surface wettability of the obtained samples, contact angle measurements were carried out. The representative photographs are depicted in Figure 7. For all of the three samples, water or phenyl acetylene drops were totally absorbed by the samples without any residue, indicating the excellent amphiphilicity of the prepared samples. Adsorption capacities of the samples for hydrophobic phenyl acetylene in water were also measured. Under the given adsorption conditions, P(QPOTf-BSA)-0.25, P(QPOTf-BSA)-0.5, and P(QPOTf-BSA)-0.75 showed high absorption capacities of 8.6, 6.5, and 4.1 g/g, respectively, as shown in Table 1. It was worth noting that the adsorption capacities were much higher than that of their pore volumes. Indeed, these high adsorption capacities could reasonably be attributed to the excellent swelling properties of the samples. Therefore, the above experimental results revealed that the prepared samples had excellent surface amphiphilicity and high adsorption capacity for hydrophobic substrates. For the solid catalyst in water, the excellent hydrophilicity enables the catalyst to disperse well in water, while the excellent lipophilicity and swelling property could enrich organic substrates in the porous framework of the catalyst, and thus accelerating the reaction rate [30]. Hence, it is not unreasonable to expect that the prepared solid could be a promising heterogeneous catalyst for the hydration of alkynes in water due to its excellent surface amphiphilicity, hierarchically porous structure, and nice swelling property.

### 3.2. Catalytic Activity

With the prepared solid acids in hand, we then tested their catalytic performances for the hydration of alkynes in water. Firstly, the hydration of phenyl acetylene was chosen as a model reaction to optimize the catalyst. As shown in Table 2, among the prepared catalysts, P(QPOTf-BSA)-0.5 showed the highest catalytic activity, affording an 83% yield of acetophenone with 10 mol% of acid dosage (entry 2). For comparison, catalytic activities of amberlyst-15, H_2_SO_4_, and PhSO_3_H were also examined (entries 4-6). The results revealed that P(QPOTf-BSA)-0.5 showed higher catalytic activity than that of these commercial solid and liquid acids. The above results indicated that P(QPOTf-BSA)-0.5 was an active solid acid for hydration of phenylacetylene in water.

**Figure 8:**
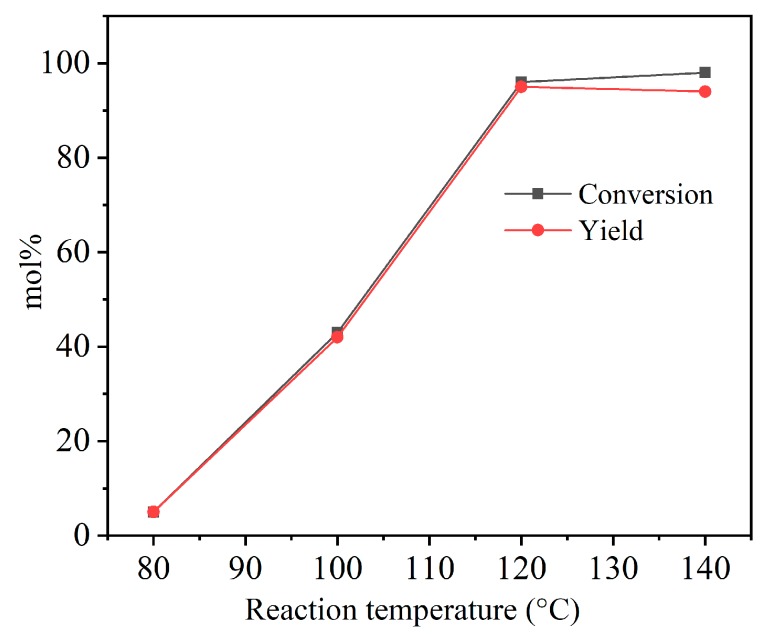
Effect of reaction temperature. Reaction conditions: phenylacetylene (2.0 mmol), P(QPOTf-BSA)-0.5 (10 mol%), H_2_O (4 mL), 120 °C, 10 h, 800 rpm.

Next, the effects of reaction parameters, including reaction temperature, catalyst loading, and reaction time, on the yield of acetophenone were investigated. It can be seen from Figure 8 that the reaction temperature had a significant effect on the hydration reaction. When the temperature varied from 80 to 120 °C, the yields of acetophenone rapidly rose from 5% to 95%. Further increasing the temperature to 140 °C led to a slight lower yield of acetophenone. Thus, 120 °C is the optimal reaction temperature for the following hydration experiments.

**Figure 9:**
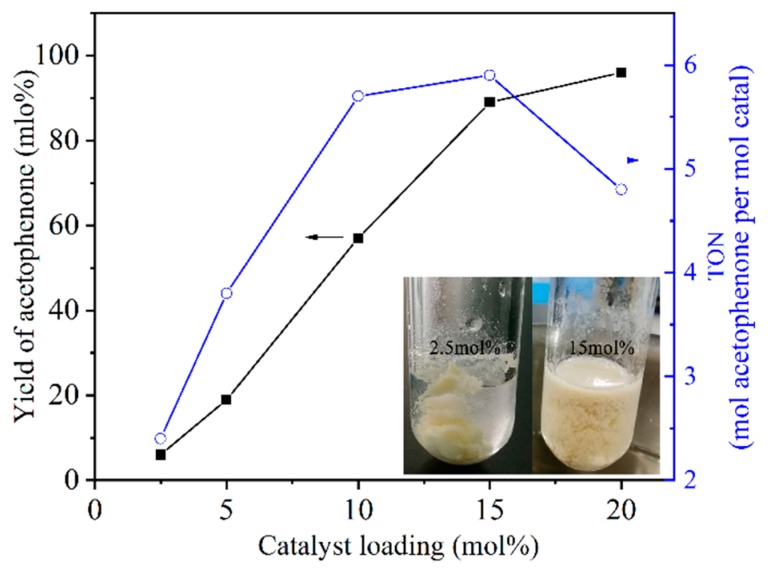
Effect of catalyst loading. Reaction conditions: phenylacetylene (2.0 mmol), P(QPOTf-BSA)-0.5, H_2_O (4 m L), 120 °C, 5 h, 800 rpm. The blue curve indicates the TON numbers, while the arrows point to the y-coordinate of the curve.

Then, the effect of catalyst loading was investigated under the optimal reaction temperature. As depicted in Figure 9, it was apparent that the yields of acetophenone were increased with the increment of acid loading, and an almost quantitative yield of acetophenone was obtained with 20 mol% of P(QPOTf-BSA)-0.5. Interestingly, the turnover numbers (TON) of P(QPOTf-BSA)-0.5 showed a volcano-type behavior, reaching the maximum yield at a 15 mol% catalyst loading. The turnover number of a 15 mol% catalyst loading was about 2.5 times higher than that of a 2.5 mol% catalyst loading. This difference could be attributed to the agglomeration of the catalyst at low catalyst loading, as seen clearly during the catalytic tests, as shown in the inset of Figure 9. With a low catalyst loading, the amphiphilic and swelling catalyst adsorbed a large number of hydrophobic substrates, thus agglomerating together to form large agglomerates. Meanwhile, the large hydrophobic agglomerates decreased the interface between the solid catalysts and water, resulting in an inferior catalytic activity. With a high catalyst loading, most hydrophobic substrates adsorbed in the inner pores of the catalyst, and the catalyst could be well dispersed in water, thus retaining a high reaction interface between water and phenylacetylene. In view of the catalytic activity, 15 mol% was taken as the optimum catalyst loading.

**Figure 10:**
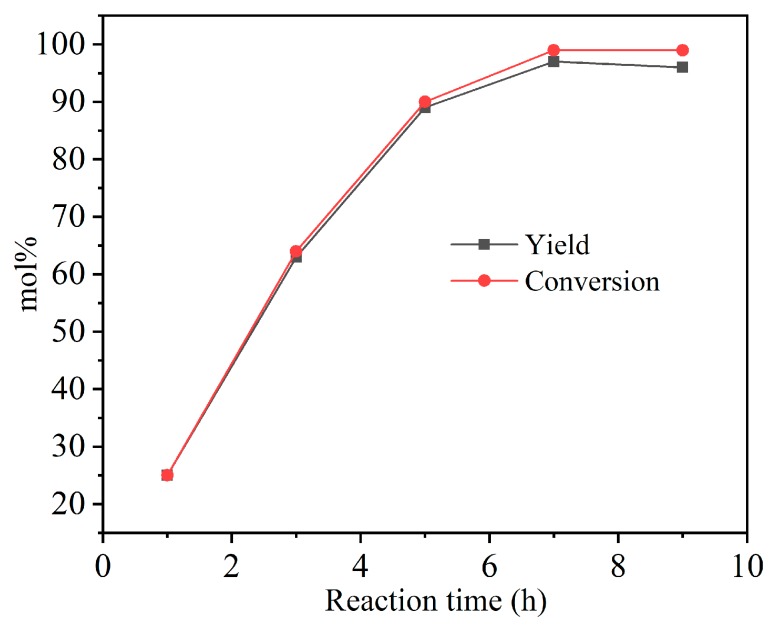
Effect of reaction time. Reaction conditions: phenylacetylene (2.0 mmol), P(QPOTf-BSA)-0.5 (H^+^, 15 mol%), H_2_O (4 mL), 120 °C, 800 rpm.

Next, the effect of the reaction time was examined. It can be seen from Figure 10 that the yield of acetophenone gradually increased to 97% within 7 h and kept no obvious change after prolonging the reaction time. For comparison, the effects of the reaction time on the catalytic activities of P(QPOTf-BSA)-0.25 and P(QPOTf-BSA)-0.75 were tested. As shown in Figure S3 from Supplementary Materials the results further verified the higher catalytic activity of P(QPOTf-BSA)-0.5.

**Figure 11:**
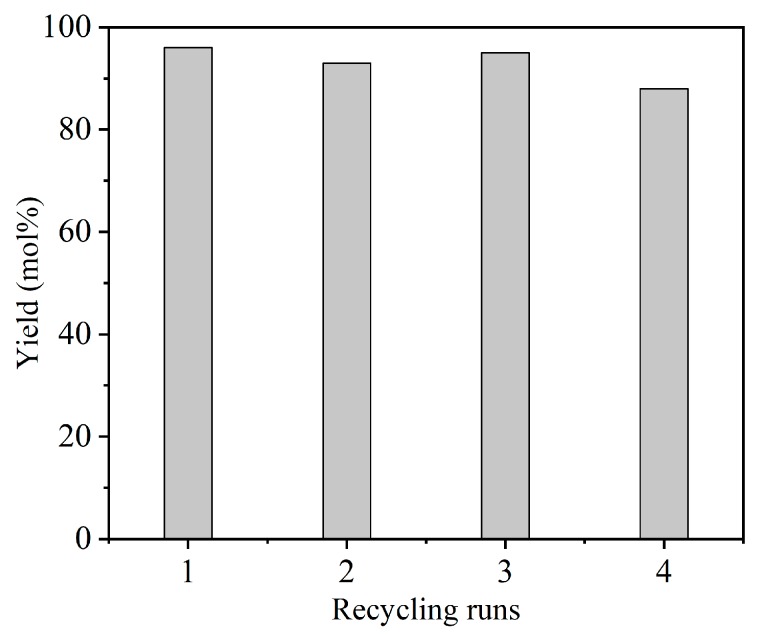
Recycling of the catalyst. Reaction conditions: phenylacetylene (2.0 mmol), P(QPOTf-BSA)-0.5 (H^+^, 15 mol%), H_2_O (4 mL), 120 °C, 7 h, 800 rpm.

**Figure 12:**
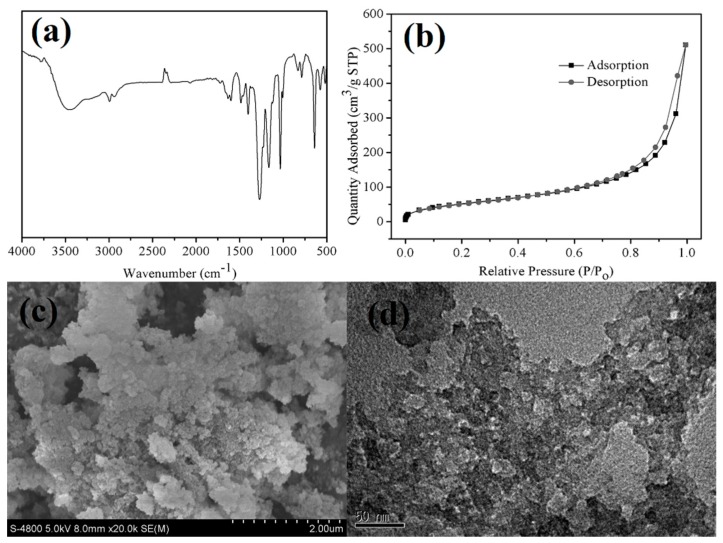
Characterization of P(QPOTf-BSA)-0.5 after four recycling tests. (**a**) FT-IR, (**b**) N_2_ absorption-desorption isotherm, (**c**) SEM, (**d**) TEM.

In addition to catalytic activity, the reusability and stability of the solid catalyst are also key influential factors for its practical application. After the completion of a reaction, the reaction mixture was separated by centrifugation. The recycled catalyst was washed with ethanol and water, and then used directly for the next catalytic run. The recycling operation was repeated four times to examine its stability. As shown in Figure 11, P(QPOTf-BSA)-0.5 showed good reusability and could be reused four times with only a slight decrease in its activity. The structure and morphology of the P(QPOTf-BSA)-0.5 after four recycling tests were studied by FT-IR, BET, SEM, and TEM. As depicted in Figure 12a, the recovered catalyst showed similar FT-IR spectrum to the fresh one, as shown in Figure 3, indicating no obvious structure change during the recycling operation. The BET result in Figure 12b suggested that a relatively big surface area of 192.3 m^2^ g^−1^ was still preserved. SEM and TEM analyses in Figure 12c,d also revealed no obvious change of morphology during the recycling process. To investigate the stability further, the acid content of the recovered P(QPOTf-BSA)-0.5 was also tested. The result suggested that the recovered P(QPOTf-BSA)-0.5 had an acid content of 1.03 mmol/g, indicating that about 88% of acid was still preserved after reusing four times.

Having the optimal reaction conditions in hand, we next explored the scope and generality of the catalytic system. As presented in Table 3, phenylacetylene substituted with various functional groups, such as 4-Me, 3-Me, 2-Me, 4-OMe, and 4-Cl, were well tolerated, affording corresponding methyl ketones in moderate to excellent yields (entries 1-5). 4-(Trifluoromethyl)phenylacetylene, an inactive substrate for hydration reactions in water [9], also reacted smoothly, and afforded an 82% yield of corresponding ketones at a prolonged reaction time (entry 6). Besides aromatic alkynes, the hydration of aliphatic alkynes was also examined. As two representative examples, 1-octyne and 4-phenyl-1-butyne could also afford corresponding methyl ketones in moderate yields (entries 7 and 8). Thus, this study offers an active and heterogeneous catalyst for the hydration of alkynes in water.

## 4. Conclusions

In summary, a porous polymer-based solid acid catalyst with excellent amphiphilicity was successfully prepared via a solvothermal copolymerization and a successive ion-exchange method. The prepared material possessed large BET surface areas, a hierarchically porous structure, and excellent amphiphilic and swelling properties. These unique properties allowed the prepared material to disperse well in water and enrich large amounts of hydrophobic alkynes in its porous frameworks. Thus, in pure water, the solid acid exhibited high catalytic performances for the hydration of alkynes, which outperformed commercial amberlyst-15, sulfuric acid, and benzenesulfonic acid. Moreover, the prepared solid acid can be easily recovered and reused at least four times. This protocol provides not only an efficient solid acid for environmentally friendly hydration of alkynes, but also some clues for the preparation of water-compatible solid acids.

## Figures and Tables

**Table 1:** Textural properties, acid content, and adsorption capabilities of the prepared samples.

Samples	*S*_BET_ (m^2^ g^−1^) *^a^*	*V*_p_ (cm^3^ g^−1^) *^b^*	D_ave_ (nm) *^c^*	Acid Content (mmol/g) ^d^	Adsorption Capability (g/g) ^e^
P(QPOTf-BSA)-0.25	392	0.56	3.2	0.45	8.6
P(QPOTf-BSA)-0.5	204	0.68	5.1	1.17	6.5
P(QPOTf-BSA)-0.75	130	0.61	8.8	2.34	4.1

*^a^* BET surface area. *^b^* Single point adsorption total pore volume of pores at *P/Po* = 0.95. *^c^* Barrett-Joyner-Halenda (BJH) adsorption average pore radius (2V/A). *^d^* Measured by acid–base titration. ^e^ Adsorption capability for phenyl acetylene in water.

**Table 2:**
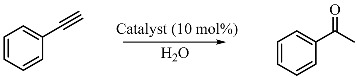
Optimization of the hydration of phenyl acetylene.

Entry	Catalyst	Conversion (mol%) ^b^	Yield (mol%) ^b^
1	P(QPOTf-BSA)-0.25	75	75
2	P(QPOTf-BSA)-0.5	84	83
3	P(QPOTf-BSA)-0.75	67	67
4	Amberlyst-15	32	30
5 ^c^	H_2_SO_4_	5	5
6	PhSO_3_H	14	13

^a^ Reaction conditions: phenylacetylene (2.0 mmol), catalyst (H^+^, 10 mol%), H_2_O (4 m L), 120 °C, 8 h, 800 rpm. ^b^ Determined by gas chromatography (GC). ^c^ H_2_SO_4_ (0.2 mmol).

**Table 3:** Hydration of various alkynes catalyzed by P(QPOTf-BSA)-0.5 ^a^.

Entry	Alkyne	Product	Yield (mol%) ^b^
1	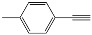	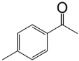	95
2	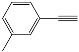	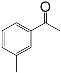	95
3	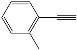	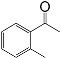	86
4	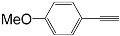	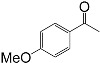	91
5	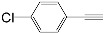	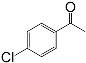	92
6 ^c^	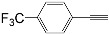	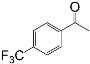	82
7 ^c^	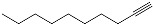	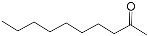	73
8 ^c^	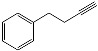	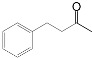	67

^a^ Reaction conditions: alkyne (2.0 mmol), P(QPOTf-BSA)-0.5 (H^+^, 15 mol%), H_2_O (4 mL), 120 °C, 7 h, 800 rpm. ^b^ GC yields. ^c^ 20 h.

## Supplementary Materials

The following are available online at https://www.mdpi.com/2073-4360/11/12/2091/s1, Figure S1: Solid-state ^31^P NMR spectrum of P(QPOTf-BSA)-0.5, Figure S2: Thermogravimetric analyses (TGA) of P(QPOTf-BSA)-0.5 sample, Figure S3: Effect of the reaction time on the catalytic activities of the P(QPOTf-BSA)-x. Reaction conditions: phenylacetylene (2.0 mmol), P(QPOTf-BSA)-x (H^+^, 15 mol%), H_2_O (4 mL), 120 °C, 800 rpm.
