# Supplementary material for: A Porous Polymer-Based Solid Acid Catalyst with Excellent Amphiphilicity: An Active and Environmentally Friendly Catalyst for the Hydration of Alkynes
Source: Polymers (Basel). 2019 Dec 13;11(12):2091. doi: 10.3390/polym11122091 (PMC6960505; doi:10.3390/polym11122091)
Supplement: Supplementary file 1 [file polymers-11-02091-s001.pdf]

polymers

Supplementary Materials

A Porous Polymer-Based Solid Acid Catalyst with Excellent Amphiphilicity: An Active and Environmentally Friendly Catalyst for the Hydration of Alkynes

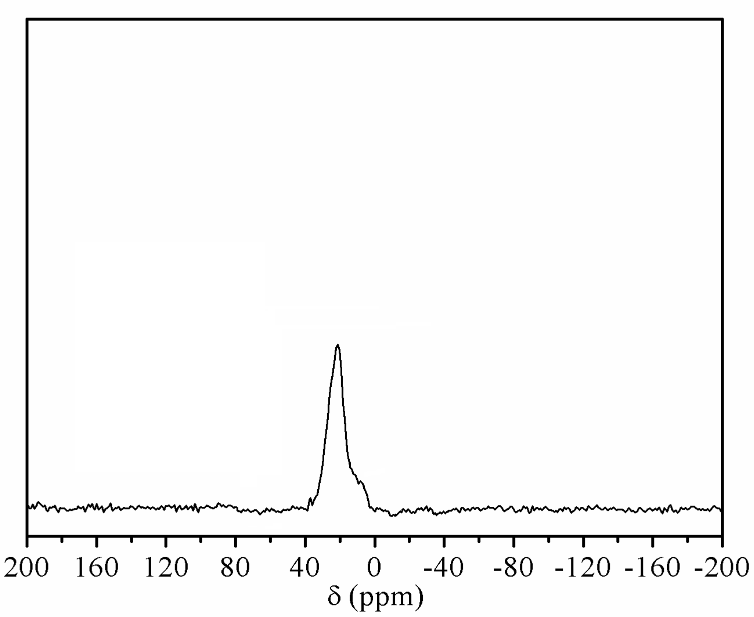

Figure S1. Solid-state ^{31}P NMR spectrum of P(QPOTf-BSA)-0.5.

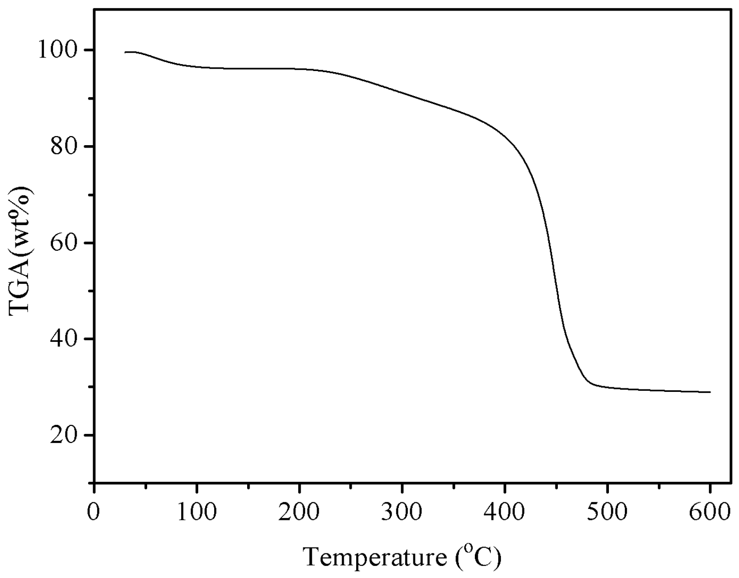

Figure S2. Thermogravimetric analyses (TGA) of P(QPOTf-BSA)-0.5 sample.

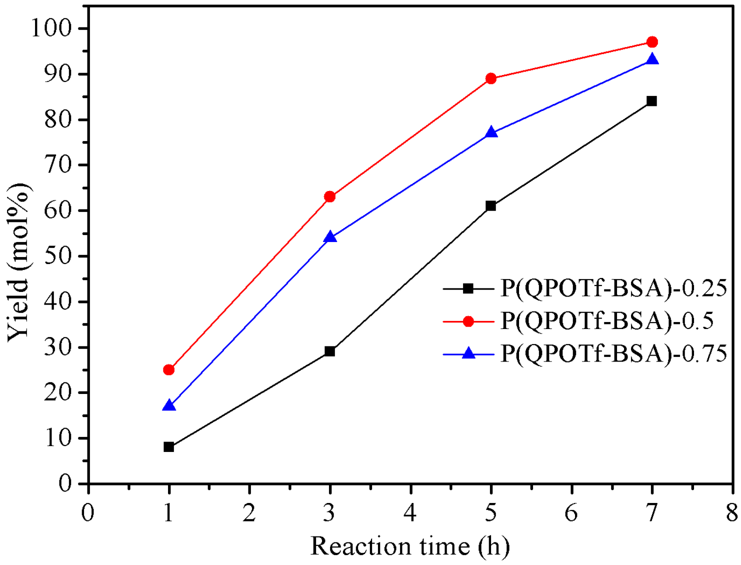

Figure S3. Effect of the reaction time on the catalytic activities of the P(QPOTf-BSA)-x. Reaction conditions: phenylacetylene (2.0 mmol), P(QPOTf-BSA)-x (H^+, 15 mol%), H_2O (4 mL), 120 °C, 800 rpm.
